# Supplementary material for: The interactome of intact mitochondria by cross-linking mass spectrometry provides evidence for coexisting respiratory supercomplexes
Source: Mol Cell Proteomics. 2017 Dec 8;17(2):216–32. doi: 10.1074/mcp.RA117.000470 (PMC5795388; doi:10.1074/mcp.RA117.000470)
Supplement: Supplemental Data [file supp_RA117.000470_133922_0_supp_23978_fzffwf.zip › spectra_annotation/mito_DR_spectra_annotation/404-1-3-1-1-1.pdf]

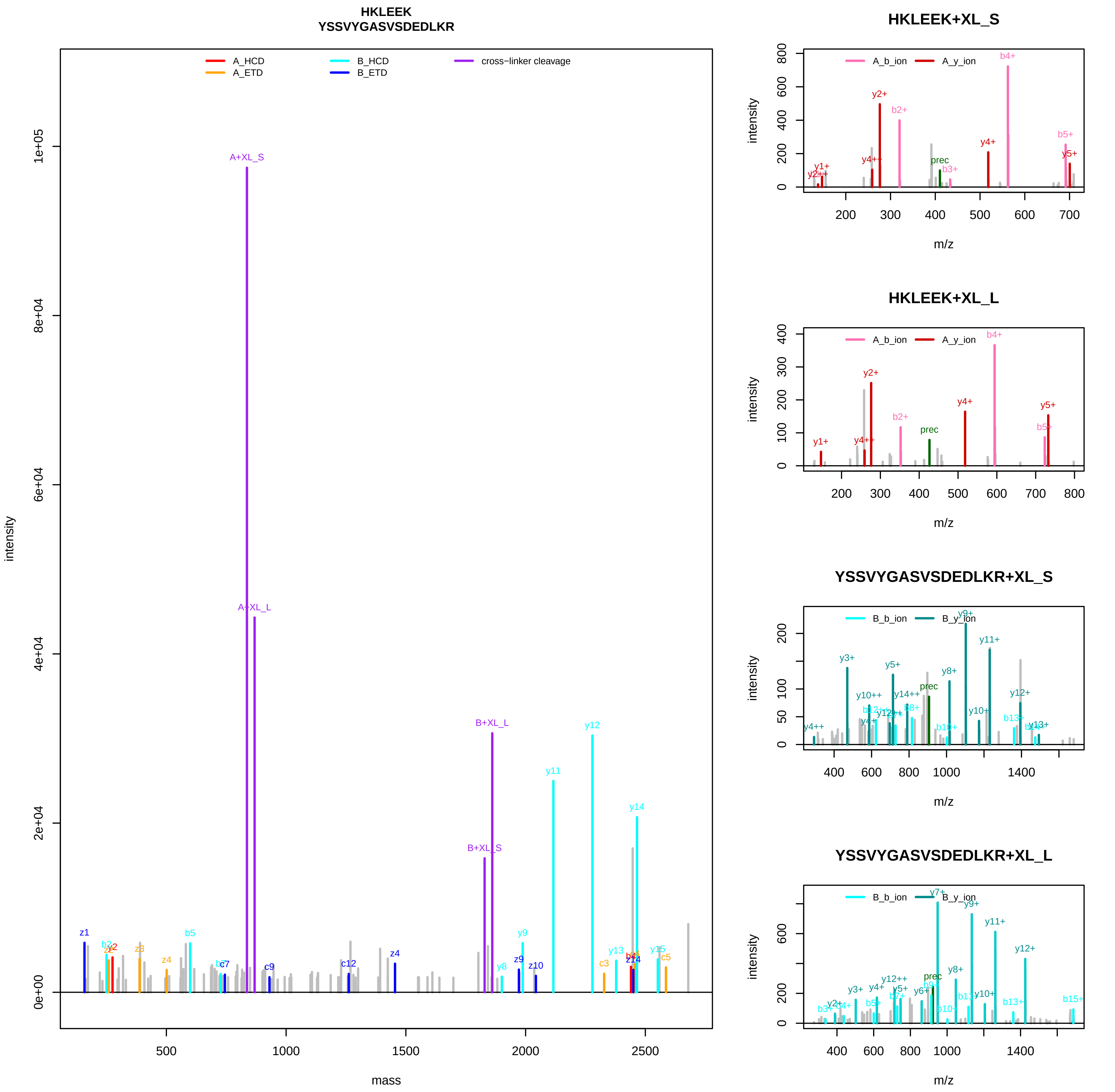
HKLEEK
YSSVYGASVSDEDLKR
A_HCD
A_ETD
B_HCD
B_ETD
cross−linker cleavage
A+XL_S
A+XL_L
B+XL_L
B+XL_S
y11
y12
y13
y14
y15
y8
y9
z1
b2
y2
z3
z4
b5
c7
c9
c12
z4
z9
z10
z14
c3
c5
intensity
mass
0e+00
2e+04
4e+04
6e+04
8e+04
1e+05
500
1000
1500
2000
2500
HKLEEK+XL_S
A_b_ion
A_y_ion
y1+
y2++
y4++
y2+
b2+
prec
b3+
y4+
b4+
b5+
y5+
m/z
HKLEEK+XL_L
YSSVYGASVSDEDLKR+XL_S
B_b_ion
B_y_ion
y3+
y5+
y8+
y9+
y10++
y14++
y10+
y11+
y12+
y13+
b13+
b10+
YSSVYGASVSDEDLKR+XL_L
y7+
y12++
y6+
b15+
